# Associations of five polymorphisms in the *CD44* gene with cancer susceptibility in Asians

**DOI:** 10.1038/srep39485

**Published:** 2016-12-21

**Authors:** Qichao Qi, Jiwei Wang, Anjing Chen, Bin Huang, Gang Li, Xingang Li, Jian Wang

**Affiliations:** 1 Department of Neurosurgery, Qilu Hospital of Shandong University and Brain Science Research Institute, Shandong University, #107 Wenhua Xi Road, Jinan, 250012, China; 2 Department of Biomedicine, University of Bergen, Jonas Lies vei 91, 5009-Bergen, Norway

## Abstract

*CD44* polymorphisms have been previously associated with cancer risk. However, the results between independent studies were inconsistent. Here, a meta-analysis was performed to systematically evaluate associations between *CD44* polymorphisms and cancer susceptibility. A comprehensive literature search conducted in PubMed, Embase, and Web of Science databases through August 10, 2016 yielded 11 eligible publications consisting of 5,788 cancer patients and 5,852 controls. Overall, odds ratios (OR) calculated with 95% confidence intervals (CI) identified a significant association between *CD44* polymorphism *rs13347* and cancer susceptibility under all genetic models. Additionally, the minor allele of polymorphism *rs11821102* was associated with a decreased susceptibility to cancer in allele contrast, dominant, and heterozygous models, while no significant association was identified for polymorphisms *rs10836347, rs713330*, or *rs1425802*. Subgroup analysis by ethnicity revealed *rs13347* was significantly associated with cancer susceptibility for Chinese but not for Indians. Linkage disequilibrium (LD) between different polymorphisms varied across diverse ethnic populations. In conclusion, the results indicate that *CD44* polymorphism *rs13347* acts as a risk factor for cancer, especially in Chinese, while the minor allele of polymorphism *rs11821102* may be associated with a decreased susceptibility to cancer. Nevertheless, further studies on a larger population covering different ethnicities are warranted.

*CD44* is a multistructural and multifunctional transmembrane glycoprotein, which functions as a receptor for hyaluronan and many other extracellular matrix components, as well as a coreceptor for growth factors and cytokines^1^. Numerous studies have demonstrated that *CD44* is involved in many crucial cellular processes, including cell survival, proliferation, differentiation, adhesion, and migration^1^,^2^.

Increasing evidence indicates that *CD44* has a role in cancer. First, *CD44* is found to be widely expressed in various cancers, and its expression correlates with poor prognosis^1^,^3^,^4^,^5^. In functional studies, specific targeted knockdown of *CD44* has been shown to impede cancer progression^6^. Second, *CD44* is a common cancer stem cell (CSC) marker. CSCs are a selected population of tumor cells that display many features of embryonic or tissue stem cells, and likewise, possess the capacity for self-renewal and differentiation. *CD44* is in fact a critical player in the regulation of properties of the CSCs, such as self-renewal, tumor initiation, metastasis, and chemo- or radio-resistance^1^,^7^. *CD44* contributes in part as a downstream target of Wnt, a gene involved in the maintenance of the CSC phenotype, and has been shown to be essential for Wnt-induced tumor progression in cancers^8^. The interaction between *CD44* and hyaluronan furthermore promotes protein kinase C activation which leads to phosphorylation and translocation to the nucleus of NANOG, a transcription factor also involved in the maintenance of stem cell characteristics^9^. Finally, *CD44* has a critical role in epithelial-mesenchymal transition (EMT). EMT is a tightly regulated and highly conserved cellular process in which a cell type changes from an epithelial to a mesenchymal phenotype^6^,^10^. In cancer, EMT is involved in the acquisition of the stemness of epithelial tumor cells, which confers an invasive phenotype onto cells that may underlie tumor recurrence and metastasis^11^,^12^.

Based on these roles in cancer development, molecular mechanisms contributing to the regulation of *CD44* and/or function have been under intensive investigation. Recent studies have examined the association of specific single nucleotide polymorphisms (SNPs) in the *CD44* gene with cancer risk but the significance of these findings remains unclear. Jiang *et al*. first discovered that the *CD44 rs13347* polymorphism might affect breast cancer development and prognosis by increasing *CD44* expression in the Chinese population^13^. Several new studies performed on Chinese also revealed a similar positive correlation with statistical power in acute myeloid leukemia, nasopharyngeal carcinoma, and colorectal cancer^14^,^15^,^16^,^17^. However, three other studies on Chinese, failed to demonstrate any statistically significant associations^18^,^19^,^20^,^21^. Furthermore, two studies conducted on Indians demonstrated that no significant association existed between *CD44 rs13347* polymorphism and risks for gallbladder or breast cancers^22^,^23^,^24^. Here, a comprehensive meta-analysis was performed to derive a more precise estimation of the relationship between *CD44* polymorphisms and the susceptibility to cancer. Eligible studies included diverse forms of cancer and two ethnicities, Chinese and Indian. These results have potential implications for the basis of cancer development as well as for functional consequences of allele specific expression of *CD44*.

## Materials and Methods

### Search strategy

PubMed, Embase, and Web of Science databases were searched for studies reporting association of *CD44* polymorphisms with cancer risk in any type of cancer performed up to the date of August 10, 2016. Gene-specific terms (*CD44*) were combined with polymorphism- (polymorphism or polymorphisms or variation or variations or variant or variants or mutation or mutations or genotype or genotypes) and disease-specific terms (cancer or cancers or tumor or tumors or neoplasm or neoplasms). The search was performed by the method of free-text word combined with the Medical Subject Headings (MeSH) and included the following search terms: “Neoplasms”, “Antigens, *CD44*” and “Polymorphism, Single Nucleotide”. A thorough review of all referenced materials within retrieved studies was also performed in order to identify additional potentially eligible studies.

### Inclusion criteria and exclusion criteria

Inclusion criteria for eligible studies were the following: (1) case-control or cohort study design; (2) sufficient data for the evaluation of *CD44* SNPs in cancer risk; (3) articles published in English; (4) studies performed on humans; and (5) in the case of multiple publications from the same study group, the most complete and recent results were used.

The exclusion criteria were defined as the following: (1) abstracts, reviews and animal studies; (2) useless reported data such as genotype number or frequency was not included; and (3) articles published in languages other than English.

### TagSNPs selection

Previous studies performed bioinformatics analysis with Haploview software 4.2 to analyze the haplotype block based on the Chinese Han Beijing population data of HapMap (HapMap Data Rel 27 Phase II + III, February 2009, on NCBI B36 assembly, dbSNP b126). Six tagSNPs were found to cover all the potential functional common SNPs (MAF > 0.05) in the *CD44* gene: *rs8193, rs11821102, rs10836347*, and *rs13347* in the 3′UTR, *rs1425802* in the promoter and *rs9666607* in exon region. *CD44 rs8193* and *rs13347* were in high linkage disequilibrium (LD) (*D*’ = 1.0, *r*^2^ = 0.527), so the selection of *rs13347* was sufficient to represent the two SNPs. In addition, due to the difficulty in genotyping *rs9666607* using MALDI-TOF, the polymorphism *rs713330*, which is in complete LD with *rs9666607 (D*’ = 1.0, *r*^2^ = 1.0) was chosen to replace it^13^,^15^,^17^.

### Data extraction

Two reviewers (Q.C.Q. and J.W.W.) independently selected studies and extracted the following data from each study: first author’s surname, publication year, ethnicity, cancer types, numbers of cases and controls, and genotype distributions of cases and controls. The results were compared and disagreement was resolved through discussion until consensus was reached. Study design was stratified into population-based studies and hospital-based studies.

### Quality assessment

The Newcastle-Ottawa Scale and Agency for Healthcare Research and Quality (http://www.ohri.ca/programs/clinical_epidemiology/oxford.asp, maximum score = 9 points) was used to evaluate the methodological quality, which scored studies by the selection of patients, the comparability of the groups, and the quality of the sampling process. Studies awarded a score of 0–3, 4–6, or 7–9 were considered as low-, moderate-, or high-quality studies, respectively.

### Statistical analysis

The meta-analysis assessed associations between polymorphisms and cancer risk under allele contrast, dominant, recessive, homozygous, and heterozygous models. Odds ratios (ORs) with 95% confidence intervals (CIs) were used to estimate the strength of associations. Heterogeneity among the included studies was evaluated using the Chi square-based Q statistic. The fixed-effect (Mantel-Haenszel method) or random-effects model (DerSimonian-Laird method) was used to calculate pooled effect estimates in the presence (*P* < 0.05) or absence (*P *> 0.05) of heterogeneity. Sensitivity analysis was conducted by sequentially excluding one study at a time. The Begg’s test and the Egger’s test were performed to assess publication bias. If any possible bias was observed, the trim and fill method was used to identify and adjust for those studies. Data analysis was carried out using Stata software, version 11.0 (Stata Corporation; College Station, TX, USA). *P*-values < 0.05 were considered to be statistically significant.

### Linkage disequilibrium (LD) analysis across populations

We extracted data from the 1000 genomes Project Phase III (https://www.ncbi.nlm.nih.gov/variation/tools/1000genomes/) regarding the *CD44* polymorphisms evaluated in the current study. Briefly, populations enrolled in the project including African ancestry in Southwest USA (ASW), Utah residents with Northern and Western European ancestry (CEU), Han Chinese in Beijing, China (CHB), Southern Han Chinese, China (CHS), Finnish in Finland (FIN), British in England and Scotland (GBR), Gujarati Indians in Houston, Texas (GIH), Indian Telugu in the UK (ITU), Japanese in Tokyo, Japan (JPT), and Toscani in Italy (TSI). Haploview software 4.2 was used to perform the analysis, and linkage disequilibrium (LD) was evaluated by *D*’ and *r*^2^ statistics in each of the above-mentioned populations^25^.

## Results

### Study characteristics

The literature search yielded 15 studies that were carefully reviewed based on the inclusion criteria^13^,^14^,^15^,^16^,^17^,^18^,^19^,^20^,^21^,^22^,^23^,^24^,^26^,^27^,^28^. Among these articles, the following studies were excluded: 2 studies that did not investigate the association between cancer risk and the polymorphism of interest^27^,^28^; 1 study that did not offer sufficient raw data^26^; and 1 study in which the data was repeated in a second publication with a larger population conducted by the same study group^22^,^24^. Thus, 11 publications were eligible for the meta-analysis for a total of 5,788 cancer patients and 5,852 controls and 9 different cancer types. The main characteristics of the eligible studies are listed in Table 1. All studies scored a value of 7 or above (high-quality) as determined in the Newcastle-Ottawa Scale, and individuals therein were of Chinese or Indian descent.

Two publications that contained the same population of controls from the same research group were considered as one case-control study^18^,^19^. The following is the breakdown of the number of studies, including cancer types, cases and controls, that met our eligibility criteria for each polymorphism evaluated: *rs13347* polymorphism, 10 case-control studies including 9 different cancer types with 5,788 cases and 5,852 controls; *rs11821102* polymorphism, 7 case-control studies including 7 different cancer types with 4,686 cases and 4,893 controls; *rs10836347* polymorphism, 6 case-control studies including 6 different cancer types with 4,411 cases and 4,618 controls; *rs713330* polymorphism, 5 case-control studies including 6 different cancer types with 3,453 cases and 3,397 controls; and *rs1425802* polymorphism, 5 case-control studies including 6 different cancer types with 3,453 cases and 3,397 controls (Table 1).

### Quantitative synthesis

**Figure 1:**
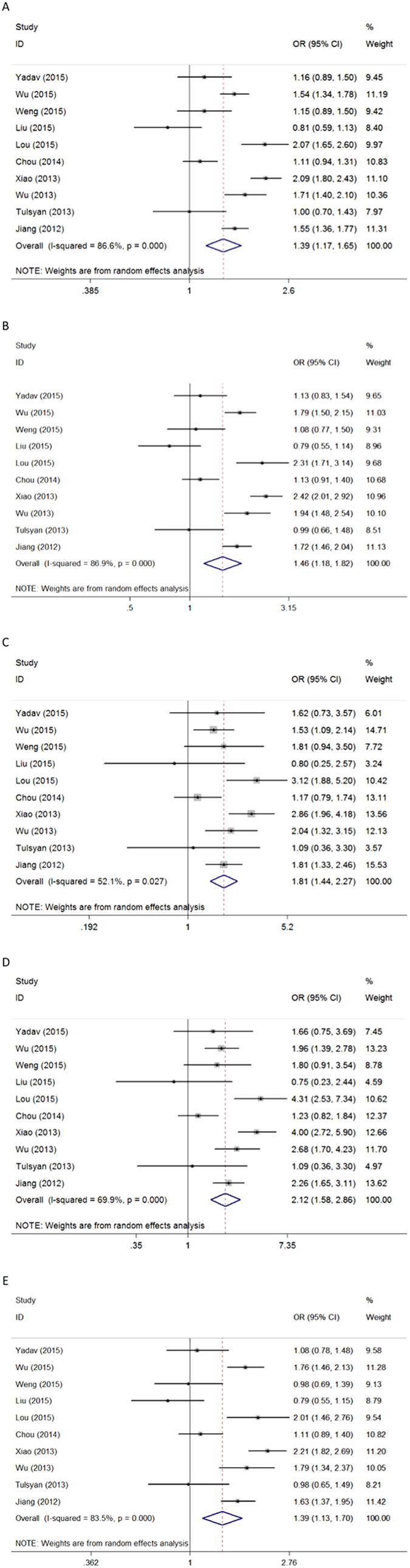
Forest plot of cancer risk associated with *CD44* polymorphism *rs13347* using different sources of controls. Models represented in (**A**) allele contrast, (**B**) dominant, (**C**) recessive, (**D**) homozygous, and (**E**) heterozygous. Each square represents a study, and the area of each square is proportional to the weight of the study. The diamond represents the summary OR and 95% CI. OR: odds ratio; CI: confidence interval.

**Figure 2:**
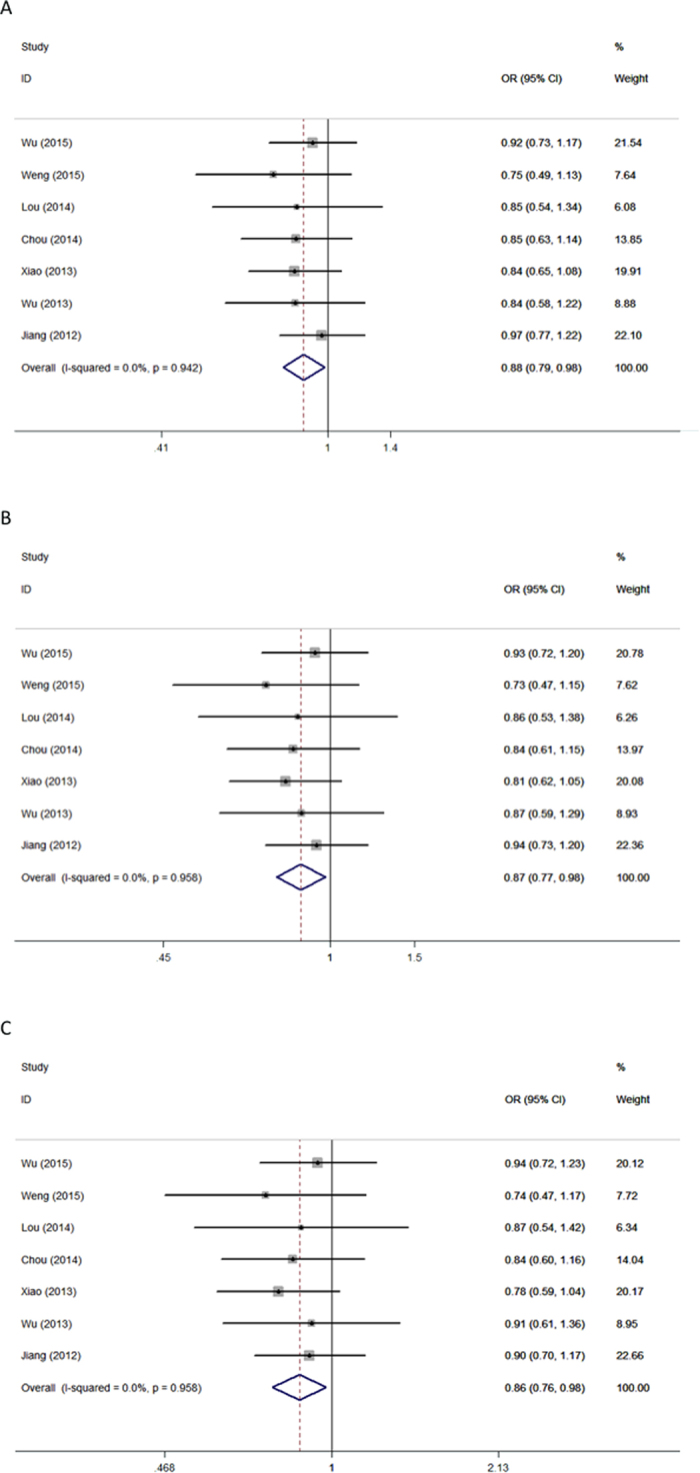
Forest plot of cancer risk associated with *CD44* polymorphism *rs11821102* in using different sources of controls. Models represented in (**A**) dominant and (**B**) heterozygous. Each square represents a study, and the area of each square is proportional to the weight of the study. The diamond represents the summary OR and 95% CI. OR: odds ratio; CI: confidence interval.

The results of association of *CD44* SNPs with a general risk for cancer including data for all individuals are shown in Table 2. Significant association between *CD44* polymorphism *rs13347* and cancer susceptibility was observed under all genetic models (Fig. 1): allele contrast (T *vs.* C, OR = 1.391, 95% CI = 1.172–1.650, *P* < 0.001); dominant model (TT+CT *vs.* CC, OR = 1.462, 95% CI = 1.176–1.818, *P* = 0.001); recessive model (TT *vs.* CT+CC, OR = 1.810, 95% CI = 1.440–2.275, *P* < 0.001); homozygous model (TT *vs.* CC, OR = 2.122, 95% CI = 1.576–2.857, *P* < 0.001); and heterozygous model (CT *vs.* CC, OR = 1.389, 95% CI = 1.133–1.702, *P* = 0.002). Additionally, polymorphism *rs11821102* was significantly associated with cancer susceptibility in the allele contrast (G *vs.* A, OR = 0.881, 95% CI = 0.789–0.983, *P* = 0.024), dominant (GG+GA *vs.* AA, OR = 0.868, 95% CI = 0.771–0.977, *P* = 0.019), and heterozygous models (GA *vs.* AA, OR = 0.864, 95% CI = 0.765–0.976, *P* = 0.019) (Fig. 2). However, no significant association was identified for polymorphisms *rs10836347, rs713330*, or *rs1425802* and overall cancer susceptibility (Table 2).

For *rs13347*, significant between-study heterogeneity was present in all genetic models; therefore, a random-effects model (Der-Simonian Laird) was used. For all other polymorphisms, no significant heterogeneity was found in any genetic model. A fixed-effect model (the Mantel-Haenszel method) was thus applied.

### Subgroup analysis

For the *rs13347* polymorphism, the studies consisted of individuals of Chinese or Indian descent. However, for other polymorphisms, individuals from all eligible studies were only of Chinese descent, and the number of studies for each cancer type was less than two. Therefore, stratified analysis on the basis of ethnicity, source of controls, and cancer type was only performed on *rs13347* (Table 3).

In an ethnicity subgroup analysis, polymorphism *rs13347* was significantly associated with cancer susceptibility for Chinese: allele contrast (T *vs.* C, OR = 1.466, 95% CI = 1.218–1.765, *P* < 0.001); dominant model (TT+CT *vs.* CC, OR = 1.571, 95% CI = 1.242–1.988, *P* < 0.001); recessive model (TT *vs.* CT+CC, OR = 1.858, 95% CI = 1.443–2.393, *P* < 0.001); homozygous model (TT *vs.* CC, OR = 2.248, 95% CI = 1.624–3.110, *P* < 0.001); and heterozygous model (CT *vs.* CC, OR = 1.482, 95% CI = 1.190–1.846, *P* < 0.001). No associations were observed between the *CD44* polymorphisms examined and Indians. The magnitude of association in hospital-based studies was significantly weakened for all genetic models. However, the magnitude of association in population-based studies was not significantly changed: allele contrast (T *vs.* C, OR = 1.607, 95% CI = 1.367–1.890, *P* < 0.001); dominant model (TT+CT *vs.* CC, OR = 1.782, 95% CI = 1.451–2.189, *P* < 0.001); recessive model (TT *vs.* CT+CC, OR = 1.945, 95% CI = 1.545–2.447, *P* < 0.001); homozygous model (TT *vs.* CC, OR = 2.489, 95% CI = 1.876–3.301, *P* < 0.001); and heterozygous model (CT *vs.* CC, OR = 1.686, 95% CI = 1.390–2.044, *P* < 0.001). When the stratification analysis was conducted based on cancer type, we uncovered that the T allele was significantly associated with an increased susceptibility to colorectal cancer, nasopharyngeal carcinoma, and acute myeloid leukemia in all genetic models, and an increased risk for breast cancer only in recessive and homozygous models.

### Sensitivity analysis

**Figure 3:**
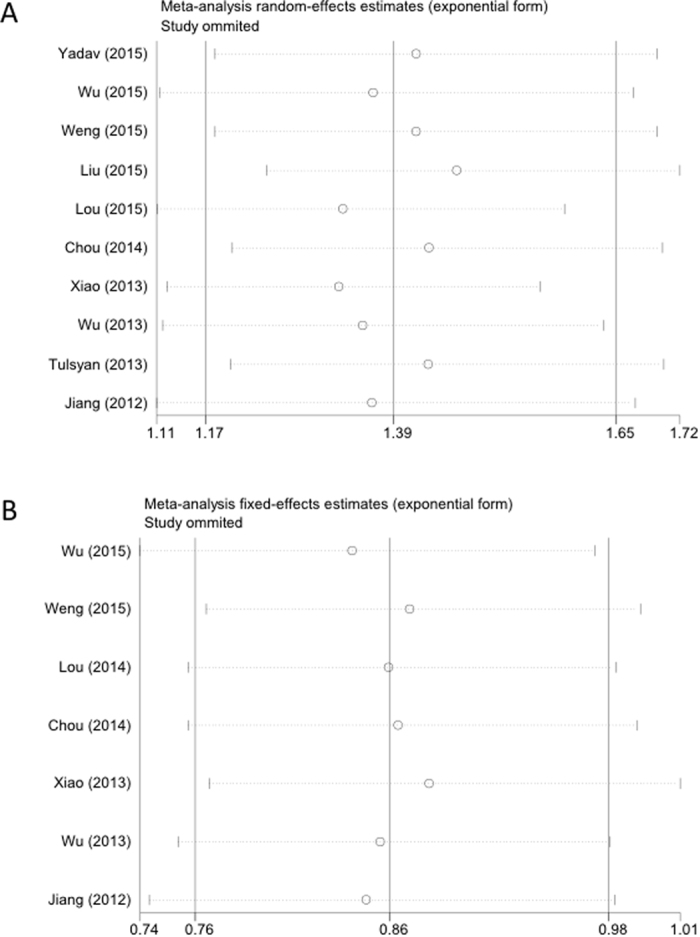
Sensitivity analysis of association between *CD44* polymorphisms and cancer risk. Polymorphisms represented in (**A**) *rs13347* under the allele contrast model and (**B**) *rs11821102* under the heterozygous model.

In order to examine the influence of individual data sets on the pooled ORs, single studies were sequentially excluded from analysis. Pooled ORs were persistent, indicating that the results were statistically stable and robust (data not shown). Sensitivity analysis of the *rs13347* polymorphism in an allelic comparison and the *rs11821102* polymorphism in the heterozygous model are presented in Fig. 3.

### Publication bias

**Figure 4:**
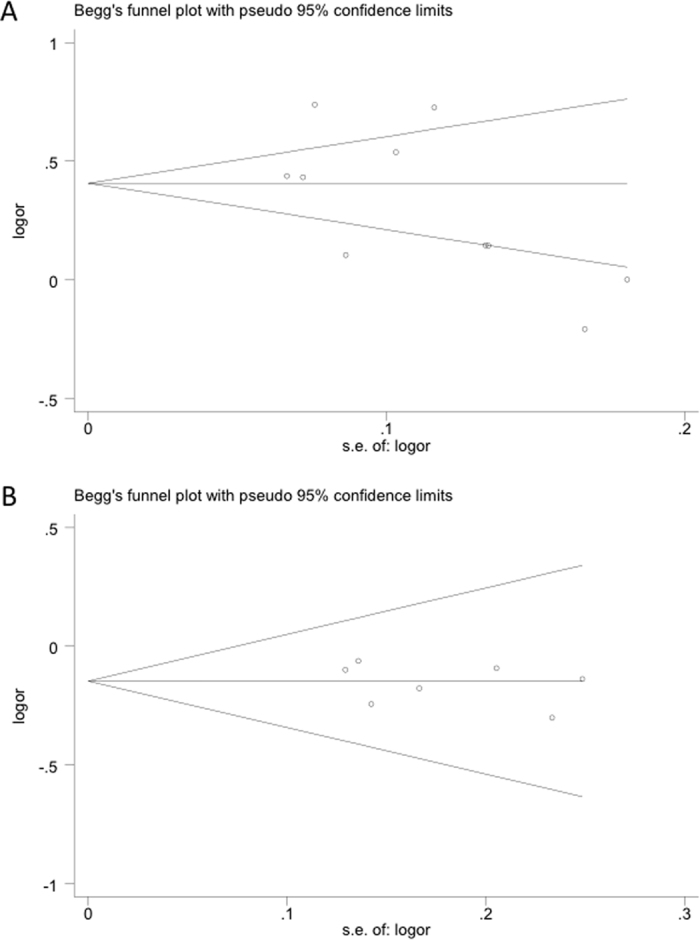
Begg’s funnel plot assessing evidence of publication bias in eligible studies used in the study. Polymorphisms represented in (**A**) *rs13347* under the allele contrast model and (**B**) *rs11821102* under the heterozygous model. The horizontal line in the figure represents the overall estimated log (OR). The two diagonal lines indicate the pseudo 95% confidence limits of the effect estimate. Log (OR): log-transformed OR; OR: odds ratio.

The Begg’s and the Egger’s tests were performed to quantitatively evaluate publication bias of the included studies. Asymmetry was not observed in the funnel plots of any of the polymorphisms. The funnel plots for the *rs13347* polymorphism in an allelic comparison and the *rs11821102* polymorphism in the heterozygous model are presented in Fig. 4, and all *P*-values from the two tests are listed in Table 2. These results were consistent with the absence of significant publication bias in the meta-analysis except in the case of polymorphism *rs10836347*. For this polymorphism, publication bias was apparent in the recessive and homozygous models. However, analysis with the trim and fill method demonstrated that the results of our study did not significantly change even after adjusting for the publication bias.

### LD analysis across populations

**Figure 5:**
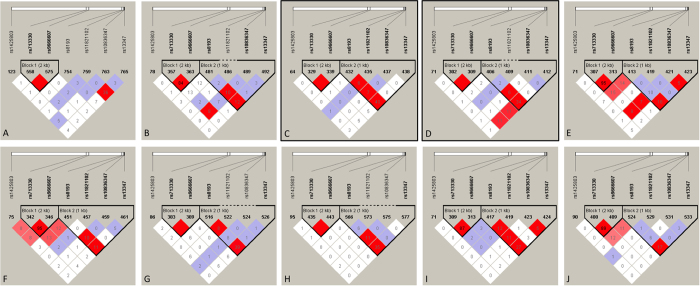
LD analyses for *CD44* polymorphisms in populations from the 1000 genomes Phase 3. The number in each cell represents the *r*^2^ value, with red, blue, and white cells representing complete LD or high LD values, moderate LD values, and low LD values or no LD between polymorphisms, respectively. Population descriptors are represented in (**A**) ASW: African ancestry in Southwest USA; (**B**) CEU: Utah residents with Northern and Western European ancestry; (**C**) CHB: Han Chinese in Beijing, China; (**D**) CHS: Southern Han Chinese, China; (**E**) FIN: Finnish in Finland; (**F**) GBR: British in England and Scotland; (**G**) GIH: Gujarati Indians in Houston, Texas; (**H**) ITU: Indian Telugu in the UK; (**I**) JPT: Japanese in Tokyo, Japan; (**J**) TSI: Toscani in Italy. The *rs* numbers are SNP IDs taken from National Center for Biotechnology Information (NCBI). LD: linkage disequilibrium; SNP: single nucleotide polymorphism.

In order to investigate the relationship between polymorphisms in the different populations, LD analysis was performed to test for the existence of bins in the region containing the seven *CD44* polymorphisms evaluated, including *rs10836347, rs11821102, rs13347, rs1425803, rs713330, rs8193*, and *rs9666607* (Fig. 5). Relatively low LD values between *rs13347* and *rs9666607* polymorphisms (*D*’ = 0.709, *r*^2^ = 0.07) were calculated for ASW populations, while complete LD (*D*’ > 0.90) was observed for the other ethnic populations examined. The *rs713330* polymorphism was in complete LD with *rs9666607 (D*’ = 1.0, *r*^2^ > 0.80) for all populations. LD plots for the ASW population yielded a high LD value between *rs13347* and *rs11821102* polymorphisms, moderate LD values for CEU, FIN, GIH, ITU, and TSI populations, and lower LD values for CHB, CHS, GBR, and JPT populations.

## Discussion

The genetic basis for the development of cancer involves inherited as well as somatic components. While familial predispositions to cancer have been more easily identified, those genetic factors more generally affecting populations in conjunction with environmental factors have been more difficult to identify. Thus, the focus has become polymorphisms in well-known cancer associated genes, such as *CD44*. The meta-analysis performed here with data pooled from 5,788 cases and 5,852 controls supports previous findings that a significant association between *CD44* polymorphism *rs13347* and cancer risk exists under all genetic models. Thus, the T allele emerged as a risk marker for cancers, especially in Chinese, which is a finding consistent with the results of most of the included studies.

The full-length *CD44* gene is located on chromosome 11 and consists of 20 exons and 19 introns^1^,^5^. Transcripts for the *CD44* gene undergo complicated alternative splicing, which results in many functionally distinct protein isoforms, such as CD44 standard (CD44s) and CD44 variant isoforms (CD44v), which account for the heterogeneity in this protein family^1^,^4^,^5^. It is widely accepted that *CD44* is expressed as multiple transcriptional variants, but the 3′UTR, which is ~3000 nucleotides long, is in fact relatively conserved^2^,^14^. The *CD44* polymorphism *rs13347* has been the subject of intense investigation in cancer susceptibility studies primarily due to its location in the 3′UTR of the *CD44* gene/transcripts^10^,^13^,^14^,^16^,^17^. The polymorphism *rs13347* may lead to alterations in mRNA and/or protein levels of *CD44* due to the change of the C and the T allele. In transient transfections using a construct in which the reporter gene is modulated by the *CD44* 3′ UTR, the presence of the *rs13347* T allele led to increased transcriptional activity relative to the C allele^13^,^15^,^16^,^17^. Similarly, by immunohistochemistry and Western blotting, carriers of the *rs13347* variant genotypes (CT and TT) were shown to exhibit dramatically increased CD44 than CC carriers in breast cancer, acute myeloid leukemia, nasopharyngeal carcinoma, and colorectal cancer^13^,^15^,^16^,^17^. Additionally, functional analyses demonstrated that hsa-mir-509–3p binds and negatively regulates the transcription of *CD44* more strongly in C allele carriers than in T allele carriers^13^,^15^,^16^,^17^. These results support previous studies that have implicated hsa-mir-509–3p as a tumor suppressor gene in the development of human cancer^29^,^30^,^31^.

In addition, when the stratification analyses were conducted based on ethnicity and source of control for the *rs13347* polymorphism, we identified a significant association in all the genetic models for Chinese but not Indians, only in population-based studies. Moreover, we performed LD analyses to determine the LD between the related polymorphisms. The *rs713330* polymorphism was in complete LD with *rs9666607* for all populations. Similarly, the high LD values between *rs13347* and *rs9666607* polymorphisms were observed in all populations except the ASW population. LD plots for the ASW population yielded a high LD value between *rs13347* and *rs11821102* polymorphisms, for CEU, FIN, GIH, ITU, and TSI populations, moderate LD values, and for CHB, CHS, GBR, and JPT populations, lower LD values. The limited number of studies and sample size may account for this discrepancy; therefore, future studies are necessary to verify these results.

None of the eligible studies for *CD44* polymorphism *rs11821102* indicate a positive correlation with statistical power associated with cancer; yet pooled analysis demonstrated that the minor allele of *rs11821102* polymorphism might be related to a decreased cancer risk in Chinese. The polymorphism *rs11821102* is also located in 3′UTR of the *CD44* gene^13^,^17^; however, no functional assay has been performed to explore how this polymorphism influences *CD44* expression or function to date. The limited number of studies and sample size may account for this discrepancy; therefore, larger, preferably population-based case-control studies, as well as mechanistic studies involving *CD44* polymorphism *rs11821102*, are warranted to validate our findings and to further investigate the role of *CD44* in the development of cancer.

Although we have conducted a comprehensive retrieval for all eligible studies and polymorphisms, which markedly expanded the sample size and revealed some associations not observed in previous work, some limitations in this study exist. First, statistically significant heterogeneity was confirmed in *rs13347* but not in other polymorphisms within all genotype models, and stratified analysis was used to explore the source of heterogeneity. Subgroup analysis by source of controls demonstrated that the between-study heterogeneity was still significant. When subgroup analysis was performed by ethnicity, the heterogeneity was dramatically reduced in Indians but not in Chinese. However, stratified analysis based on cancer type demonstrated that the heterogeneity was not significant in studies performed on nasopharyngeal carcinoma under all genetic models or on breast cancer under recessive and homozygous models. Therefore, the between-study heterogeneity might be mainly attributed to various cancer types. Second, our analysis was limited to individuals of Asian descent, mainly Chinese. LD between different polymorphisms varied across diverse ethnic populations. It, therefore, remains uncertain as to whether the result can be generalized to other ethnic populations.

To the best of our knowledge, this is the first meta-analysis to date to investigate the associations of *CD44* polymorphisms with cancer risk. In summary, our study suggests *CD44* polymorphism *rs13347* can serve as a risk factor for cancer, particularly in Chinese. Our findings also indicate that the minor allele of *rs11821102* polymorphism may be associated with a decreased susceptibility to cancer in Chinese.

## Additional Information

**How to cite this article**: Qi, Q. *et al*. Associations of five polymorphisms in the *CD44* gene with cancer susceptibility in Asians. *Sci. Rep.* **6**, 39485; doi: 10.1038/srep39485 (2016).

**Publisher's note:** Springer Nature remains neutral with regard to jurisdictional claims in published maps and institutional affiliations.

## Figures and Tables

**Table 1:** Characteristics of studies on association between *CD44* single nucleotide polymorphisms and cancers.

Single Nucleotide Polymorphisms	Author	Year	Ethnicity	Cancers	Genotyping Method	Quality Score	Cases	Controls	Source of Controls	*P* for HWE in controls	Cases			Controls		
											AA	AB	BB	AA	AB	BB
*rs13347*	Yadav^a^	2015	Indian	GBC	TaqMan, ARMS-PCR, PCR-RFLP	9	610	250	PB	0.62	378	201	31	162	80	8
*rs13347*	Wu	2015	Chinese	CRC	MALDI-TOF mass spectrometry	9	946	989	PB	0.28	416	441	89	578	348	63
*rs13347*	Weng	2015	Chinese	TCC	TaqMan	8	275	275	HB	0.15	138	111	26	143	117	15
*rs13347*	Liu	2015	Chinese	NSCLC	Taqman	8	234	468	HB	0.82	179	51	4	337	121	10
*rs13347*	Sharma^a^	2014	Indian	GBC	TaqMan, PCR-RFLP	9	405	200	PB	0.57	293	104	8	154	42	4
*rs13347*	Lou	2014	Chinese	NPC	ABI 3730xl sequencing platform	8	287	507	HB	0.91	104	126	42	288	174	27
*rs13347*	Chou-1^b^	2014	Chinese	OSCC	TaqMan	7	599	561	HB	0.92	287	262	50	295	223	43
*rs13347*	Chou-2^b^	2014	Chinese	HCC	TaqMan	7	203	561	HB	0.92	110	72	21	295	223	43
*rs13347*	Xiao	2013	Chinese	NPC	MALDI-TOF mass spectrometry	9	906	943	PB	0.64	386	418	102	606	297	40
*rs13347*	Wu	2013	Chinese	AML	MALDI-TOF mass spectrometry	9	421	461	PB	0.34	163	196	62	254	171	36
*rs13347*	Tulsyan	2013	Indian	BC	ABI 7500 Real Time PCR system	8	258	241	HB	0.58	191	60	7	178	57	6
*rs13347*	Jiang	2012	Chinese	BC	MALDI-TOF mass spectrometry	9	1049	1157	PB	0.84	451	484	114	654	430	73
*rs11821102*	Wu	2015	Chinese	CRC	MALDI-TOF mass spectrometry	9	946	989	PB	<0.001	815	119	12	843	131	15
*rs11821102*	Weng	2015	Chinese	TCC	TaqMan	8	275	275	HB	0.92	234	39	2	222	50	3
*rs11821102*	Lou	2014	Chinese	NPC	ABI 3730xl sequencing platform	8	287	507	HB	0.35	252	27	1	439	54	3
*rs11821102*	Chou-1^b^	2014	Chinese	OSCC	TaqMan	7	599	561	HB	0.28	531	63	5	481	75	5
*rs11821102*	Chou-2^b^	2014	Chinese	HCC	TaqMan	7	203	561	HB	0.28	173	29	1	481	75	5
*rs11821102*	Xiao	2013	Chinese	NPC	MALDI-TOF mass spectrometry	9	906	943	PB	0.14	796	100	10	805	129	9
*rs11821102*	Wu	2013	Chinese	AML	MALDI-TOF mass spectrometry	9	421	461	PB	0.28	370	50	1	398	59	4
*rs11821102*	Jiang	2012	Chinese	BC	MALDI-TOF mass spectrometry	9	1049	1157	PB	0.22	912	125	12	997	151	9
*rs10836347*	Wu	2015	Chinese	CRC	MALDI-TOF mass spectrometry	9	946	989	PB	0.10	821	120	5	851	129	9
*rs10836347*	Lou	2014	Chinese	NPC	ABI 3730xl sequencing platform	8	287	507	HB	0.85	249	27	2	438	55	2
*rs10836347*	Chou-1^b^	2014	Chinese	OSCC	TaqMan	7	599	561	HB	0.15	522	73	4	487	69	5
*rs10836347*	Chou-2^b^	2014	Chinese	HCC	TaqMan	7	203	561	HB	0.15	180	23	0	487	69	5
*rs10836347*	Xiao	2013	Chinese	NPC	MALDI-TOF mass spectrometry	9	906	943	PB	0.31	785	118	3	792	147	4
*rs10836347*	Wu	2013	Chinese	AML	MALDI-TOF mass spectrometry	9	421	461	PB	0.93	364	55	2	404	55	2
*rs10836347*	Jiang	2012	Chinese	BC	MALDI-TOF mass spectrometry	9	1049	1157	PB	0.97	906	139	4	995	156	6
*rs713330*	Weng	2015	Chinese	TCC	TaqMan	8	275	275	HB	0.87	231	42	2	223	49	3
*rs713330*	Chou-1^b^	2014	Chinese	OSCC	TaqMan	7	599	561	HB	0.09	507	88	4	467	86	8
*rs713330*	Chou-2^b^	2014	Chinese	HCC	TaqMan	7	203	561	HB	0.09	167	36	0	467	86	8
*rs713330*	Xiao	2013	Chinese	NPC	MALDI-TOF mass spectrometry	9	906	943	PB	0.74	732	164	10	751	180	12
*rs713330*	Wu	2013	Chinese	AML	MALDI-TOF mass spectrometry	9	421	461	PB	0.39	341	74	6	371	87	3
*rs713330*	Jiang	2012	Chinese	BC	MALDI-TOF mass spectrometry	9	1049	1157	PB	0.39	865	172	12	950	194	13
*rs1425802*	Weng	2015	Chinese	TCC	TaqMan	8	275	275	HB	0.001	105	121	49	99	109	67
*rs1425802*	Chou-1^b^	2014	Chinese	OSCC	TaqMan	7	599	561	HB	< 0.001	197	249	153	194	235	132
*rs1425802*	Chou-2^b^	2014	Chinese	HCC	TaqMan	7	203	561	HB	< 0.001	70	75	58	194	235	132
*rs1425802*	Xiao	2013	Chinese	NPC	MALDI-TOF mass spectrometry	9	906	943	PB	0.11	270	450	186	299	442	202
*rs1425802*	Wu	2013	Chinese	AML	MALDI-TOF mass spectrometry	9	421	461	PB	0.08	126	204	91	122	248	91
*rs1425802*	Jiang	2012	Chinese	BC	MALDI-TOF mass spectrometry	9	1049	1157	PB	0.55	316	513	220	353	563	241

PB: population-based. HB: hospital-based. PCR: polymerase chain reaction. RFLP: restriction fragment length polymorphism. ARMS: amplification refractory mutation system. HWE: Hardy-Weinberg equilibrium. GBC: gallbladder cancer. CRC: colorectal cancer. TCC: transitional cell carcinoma of urinary bladder. NSCLC: non-small cell lung cancer. NPC: nasopharyngeal carcinoma. OSCC: oral squamous cell carcinoma. HCC: hepatocellular carcinoma. AML: acute myeloid leukemia. BC: breast cancer.

^a^Containing repeated data.

^b^Containing the same population of controls.

**Table 2:** ORs and 95% CI for cancers and *CD44* single nucleotide polymorphisms under different genetic models.

Genetic models	N	OR [95% CI]	*P*_(OR)_	Model (method)	*I*-square (%)	*P*_(H)_	*P*_(Begg)_	*P*_(Egger)_
*rs13347*								
Allele contrast	10	**1.391 [1.172–1.650]**	**<0.001**	R (D-L)	86.6	<0.001	0.210	0.117
Dominant model	10	**1.462 [1.176–1.818]**	**0.001**	R (D-L)	86.9	**<**0.001	0.210	0.081
Recessive model	10	**1.810 [1.440–2.275]**	**<0.001**	R (D-L)	52.1	0.027	0.371	0.563
Homozygous model	10	**2.122 [1.576–2.857]**	**<0.001**	R (D-L)	69.9	**<**0.001	0.210	0.374
Heterozygous model	10	**1.389 [1.133–1.702]**	**0.002**	R (D-L)	83.5	**<**0.001	0.283	0.053
*rs11821102*								
Allele contrast	7	**0.881 [0.789–0.983]**	**0.024**	F (M-H)	0.0	0.942	0.368	0.074
Dominant model	7	**0.868 [0.771–0.977]**	**0.019**	F (M-H)	0.0	0.958	0.230	0.192
Recessive model	7	0.944 [0.625–1.427]	0.785	F (M-H)	0.0	0.821	0.133	0.088
Homozygous model	7	0.927 [0.613–1.401]	0.720	F (M-H)	0.0	0.822	0.133	0.081
Heterozygous model	7	**0.864 [0.765–0.976]**	**0.019**	F (M-H)	0.0	0.958	0.548	0.459
*rs10836347*								
Allele contrast	6	0.923 [0.822–1.035]	0.172	F (M-H)	0.0	0.865	1.000	0.423
Dominant model	6	0.927 [0.821–1.047]	0.223	F (M-H)	0.0	0.843	1.000	0.571
Recessive model	6	0.733 [0.413–1.303]	0.290	F (M-H)	0.0	0.935	**0.024**	**0.017**
Homozygous model	6	0.728 [0.410–1.294]	0.279	F (M-H)	0.0	0.936	**0.024**	**0.017**
Heterozygous model	6	0.936 [0.827–1.060]	0.299	F (M-H)	0.0	0.823	1.000	0.719
*rs713330*								
Allele contrast	5	0.942 [0.841–1.056]	0.307	F (M-H)	0.0	0.919	0.462	0.374
Dominant model	5	0.942 [0.833–1.066]	0.344	F (M-H)	0.0	0.970	0.462	0.193
Recessive model	5	0.871 [0.550–1.379]	0.555	F (M-H)	4.7	0.380	0.806	0.885
Homozygous model	5	0.863 [0.544–1.367]	0.529	F (M-H)	3.7	0.386	0.806	0.873
Heterozygous model	5	0.948 [0.835–1.076]	0.406	F (M-H)	0.0	0.968	0.221	0.217
*rs1425802*								
Allele contrast	5	1.007 [0.941–1.077]	0.849	F (M-H)	0.0	0.485	0.462	0.188
Dominant model	5	1.013 [0.914–1.122]	0.811	F (M-H)	0.0	0.627	0.462	0.139
Recessive model	5	1.003 [0.894–1.126]	0.954	F (M-H)	28.1	0.234	0.806	0.456
Homozygous model	5	1.009 [0.883–1.152]	0.900	F (M-H)	0.0	0.459	0.221	0.173
Heterozygous model	5	1.014 [0.909–1.132]	0.801	F (M-H)	0.0	0.503	0.806	0.429

OR: odds ratio. CI: confidence intervals. N: number of included studies. R: random-effects model. D-L: DerSimonian-Laird method. F: fixed-effect model. M-H: Mantel-Haenszel method. *P*_(H)_: *P* for heterogeneity. *P*-values < 0.05 were considered as statistically significant and are highlighted in bold font in the table.

**Table 3:** Subgroup analyses for *CD44 rs13347* and cancers under different genetic models.

Genetic models	N	OR [95% CI]	*P*_(OR)_	Model (method)	*I*-square (%)	*P*_(H)_	*P*_(Begg)_	*P*_(Egger)_
Allele contrast								
Overall	10	**1.391 [1.172–1.650]**	**<0.001**	R (D-L)	86.6	**<**0.001	0.210	0.117
Chinese	8	**1.466 [1.218–1.765]**	**<0.001**	R (D-L)	87.8	**<**0.001	—	—
Indian	2	1.100 [0.891**–**1.357]	0.376	R (D-L)	0.0	0.523	—	—
PB	5	**1.607 [1.367–1.890]**	**<0.001**	R (D-L)	78.7	0.001	—	—
HB	5	1.181 [0.869**–**1.605]	0.288	R (D-L)	86.3	**<**0.001	—	—
NPC	2	**2.085 [1.841–2.361]**	**<0.001**	R (D-L)	0.0	0.934	—	—
BC	2	1.287 [0.844**–**1.965]	0.242	R (D-L)	80.5	0.024	—	—
Dominant model								
Overall	10	**1.462 [1.176–1.818]**	**0.001**	R (D-L)	86.9	**<**0.001	0.210	0.081
Chinese	8	**1.571 [1.242–1.988]**	**<0.001**	R (D-L)	87.7	**<**0.001	—	—
Indian	2	1.076 [0.844**–**1.373]	0.554	R (D-L)	0.0	0.610	—	—
PB	5	**1.782 [1.451–2.189]**	**<0.001**	R (D-L)	78.7	0.001	—	—
HB	5	1.182 [0.835**–**1.673]	0.347	R (D-L)	83.7	**<**0.001	—	—
NPC	2	**2.392 [2.040–2.806]**	**<0.001**	R (D-L)	0.0	0.802	—	—
BC	2	1.348 [0.786**–**2.311]	0.277	R (D-L)	84.0	0.012	—	—
Recessive model								
Overall	10	**1.810 [1.440–2.275]**	**<0.001**	R (D-L)	52.1	0.027	0.371	0.563
Chinese	8	**1.858 [1.443–2.393]**	**<0.001**	R (D-L)	60.8	0.013	—	—
Indian	2	1.417 [0.745**–**2.697]	0.288	R (D-L)	0.0	0.570	—	—
PB	5	**1.945 [1.545–2.447]**	**<0.001**	R (D-L)	38.4	0.165	—	—
HB	5	1.562 [0.948**–**2.573]	0.080	R (D-L)	63.7	0.026	—	—
NPC	2	**2.954 [2.181–4.001]**	**<0.001**	R (D-L)	0.0	0.787	—	—
BC	2	**1.746 [1.300–2.346]**	**<0.001**	R (D-L)	0.0	0.388	—	—
Homozygous model								
Overall	10	**2.122 [1.576–2.857]**	**<0.001**	R (D-L)	69.9	**<**0.001	0.210	0.374
Chinese	8	**2.248 [1.624–3.110]**	**<0.001**	R (D-L)	74.6	**<**0.001	—	—
Indian	2	1.437 [0.752**–**2.748]	0.273	R (D-L)	0.0	0.543	—	—
PB	5	**2.489 [1.876–3.301]**	**<0.001**	R (D-L)	55.7	0.061	—	—
HB	5	1.642 [0.877**–**3.074]	0.121	R (D-L)	75.9	0.002	—	—
NPC	2	**4.106 [3.002–5.617]**	**<0.001**	R (D-L)	0.0	0.827	—	—
BC	2	**1.917 [1.050–3.501]**	**0.034**	R (D-L)	35.6	0.213	—	—
Heterozygous model								
Overall	10	**1.389 [1.133–1.702]**	**0.002**	R (D-L)	83.5	**<**0.001	0.283	0.053
Chinese	8	**1.482 [1.190–1.846]**	**<0.001**	R (D-L)	84.3	**<**0.001	—	—
Indian	2	1.040 [0.808**–**1.339]	0.758	R (D-L)	0.0	0.727	—	—
PB	5	**1.686 [1.390–2.044]**	**<0.001**	R (D-L)	73.4	0.005	—	—
HB	5	1.124 [0.833**–**1.518]	0.445	R (D-L)	76.1	0.002	—	—
NPC	2	**2.152 [1.821–2.544]**	**<0.001**	R (D-L)	0.0	0.613	—	—
BC	2	1.312 [0.801**–**2.150]	0.281	R (D-L)	79.5	0.027	—	—

OR: odds ratio. CI: confidence intervals. N: number of included studies. R: random-effects model. D-L: DerSimonian-Laird method. *P*_(H)_: *P* for heterogeneity. PB: population-based. HB: hospital-based. NPC: nasopharyngeal carcinoma. BC: breast cancer. *P*-values < 0.05 were considered as statistically significant and are highlighted in bold font in the table.
